# High Temperature Preceding Abortion

**Published:** 1885-06

**Authors:** 


### Selections.

### High Temperature Preceding Abortion.

In the *Medical Record* of May 9th, Dr. M. Howard Russell, of Philadelphia, reports two cases of high temperature preceding abortion, one rising to 105.4° the other to 103.5°. In the experience of most observing general practitioners, the occurrence of high temperature before abortion is not uncommon, when there are even no pains, or hemorrhage, or other precursory signs or symptoms. A severe chill with sudden high rise of temperature in a woman known to be pregnant, especially when no adequate cause can be found for the condition, besides, should be looked upon as a possible and almost probable harbinger of an abortion to follow. Being thus forewarned he may by proper treatment avert the abortion. Dr. Russell concludes thus: "These two cases illustrate well the purely nervous origin of some febrile conditions.

